# New Jersey State Dental Society

**Published:** 1883-10

**Authors:** 


### NEW JERSEY STATE DENTAL SOCIETY.

(Continued from page 489, Sept. No.)

AFTERNOON SESSION, JULY 19th.

Dr. Geo. C. Brown read a paper entitled the different preparations of gold for filling teeth.

This paper has not been received.—EDITOR.

*Dr. Geran.*—I like the tone of the paper. It is a sort of register, and carries us back to the time when dentistry was in its infancy ; it shows that we are a progressive profession, and I know of no other that has made the progress that this our beloved dental profession has made. Why is that so? It is because we have teachers that are leading us on higher and higher, and will continue to lead us upward.

*Dr. Bodecker.*—For my part I thank Dr. Brown very much for his interesting paper, but I think the remarks made in it with reference to soft foil are not altogether correct. Very many gentlemen profess to use soft foil, but when you examine the manner in which they introduce it into the cavity, you will find that they are making it cohesive. Almost every one will carry the gold through the flame of a spirit lamp before placing it in the cavity, and gold that has been treated in that manner cannot, strictly speaking, be called soft foil. In using soft foil as such it ought not to be touched by the annealing flame at all. Soft foil, when it is put against the walls of the cavity without annealing, makes a beautiful filling, but as to the outer surface I think cohesive foil is far more serviceable.

*Dr. Atkinson.*—Perhaps our differences in regard to gold may be due to a false denomination. Cohesive gold is the softest possible condition of gold. The non-cohesive gold is non-cohesive because of some foreign substance on its surface, or because of a harder film. With that exception, I too thank Dr. Brown for the beautiful excerpt of the history of the use of gold that he has brought before us to-day. As to the little fling about birds flying high and lighting low, that depends upon whether their wings are clipped. Heavy gold has not had its wings clipped. It has not been deprived of the power to make a filling in less time and more solidly than any other form of gold. If I want to do the very best work, I want at least No. 240 gold, and if the cavity is small and the walls very strong, I want 480. You will learn that the strength of the walls of your cavity and the resistance they will afford has a philosophical bearing on this question.

*Dr. J. A. Robinson.*—When I began practice, forty-seven years ago, it was with Bull's soft foil. At that time I was in Boston, and we knew nothing about the cohesive properties of gold, and having learned the old way, and having tried to keep up with the new way, I have formed the practice of placing soft foil to the walls of my cavities and then finishing with cohesive. Although I have no absolute means of knowing whether the Abbey foil would cohere if passed through the flame of a lamp, as the cohesive foil does to-day, yet I hardly think it would cohere like crystal gold. But when we take into consideration the fact that gold raised to 400 degrees Fahrenheit throws out crystals that have been beaten down in the process of manufacture between the gold-beater's skins, until they have lost the *grabs* that naturally cover the surface and that make them cohere to other pieces when brought in contact, then we have made the first step in the process toward a solid filling. I think it makes very little difference whether you use No. 60 or 120, or No. 4 folded so many times as to make that thickness, because after the filling is condensed you have substantially the same thing. I do believe, from what I have seen in my days and years of observation, that the dentistry of the former time, of Harwood and Tucker, was, for hard service, nothing to compare with what we have to-day. That was partly because of the theory that prevailed at that time, and which was taught to me when I began practice, that we ought not to make experiments. They said : Do not try to do anything that you are not sure of accomplishing, because if you should happen to fail you will lose caste. Attempt only that which will give you credit. Take no chances. Dead men tell no tales. All teeth that you are not sure of saving pull out and cast them from you. That was the theory, and I was taught with the forceps to take hold of everything that was a little doubtful and pluck it out. They said : You cannot save all the teeth, and if you can save ten or fifteen of them by filling the small cavities, you have done the greatest good that you can possibly do. Now when we extract teeth,—well, we don't extract now at all; we take the old roots that we used to pull out years ago and build them up with gold, or supply them with artificial crowns, and I have teeth on both the lower and upper jaw placed over roots that were tolerably sound, that I have worn artificial dentures over for more than twenty years. So instead of extracting teeth that are pretty bad as we used to do, and that could have been saved had we known enough, we leave them in, and place serviceable artificial crowns on them ; and so we go on from glory to glory, until we shall stand before the golden gate and see the golden city, and say : Praises be to the Lord forever.

*Dr. Watkins.*—Dr. Robinson gave us a beautiful description of his work, and I think it would be well for him to tell how he does it. He secures some of that saving of old teeth with a material that is called Robinson's textile fibrous metallic filling, I believe.

*Dr. Robinson.*—I will not detain you long, because Dr. Atkinson told me the best plan was to be modest and follow his lead; and that is true.

Being satisfied that soft gold, with my own manipulation, would more thoroughly stop cavities in the cervical walls and in the buccal and lingual surfaces of teeth than anything else I could use, I used it. Then, when I found myself disappointed sometimes, and saw a hole burrowing under the filling, I said that something that would so stop this cavity and hermetically seal it up that it would not leak, was what I wanted and would try to find. I made a great many experiments to obtain a fibrous material that would recoil within itself, so that the filling would not retreat from the borders or margins. When a fibrous metal has got no recoil to it it stays where you place it. These fibers do not have any possible connection one with the other, only under pressure, but when placed in a cavity, either by hand pressure or the mallet, the wall is hermetically sealed at the bottom, and it bears the same relation, in my opinion, that the grouting does to the wet places in the substructure of the building, and is better than solid granite to hold the superstructure. Having filled the greater part of the cavity with this material, I then put the gold upon it, which coheres; not exactly coheres, but adheres. The interlocking and interlacing of the gold with the fibrous material makes the crystals of gold and the crystals of the fibrous material interlock until it is so united that you cannot separate the gold from the fibrous material, and that I call Robinson's fibrous and textile metallic filling. This combination of materials makes a better filling than can otherwise be made, whether you use fine foil or thick foil. When it is covered with the gold the latter bears the same relation to the filling that the steel facing of the hammer bears to the iron hammer, and you have the same qualities that you would have had with gold alone, and the filling is made in a quarter or an eighth part of the time that would be required to do it in any other way, and you have a better result. This material is not a plastic material ; it is fibrous, and adhesive by being interlocked, and the gold is interlocked with it in the same way. This is somewhat new here, although it is not new in Chicago and Detroit, where it has been in use for three years. I find it harder to introduce things at the east than it is at the west. Perhaps the western men are more pliant; they are not all adamant, and you can make an impression on a Chicago man quicker by one hundred per cent than you can on a Boston man. I found in the dental offices in Boston men who claim to be good operators of the old soft foil type, who had never had a rubber dam on a tooth and never put rubber in the mouth, and who say they would not have it. I also found men who claimed to be good operators who have never used a dental engine, but who continue to operate in the primitive way of fifty years ago. What we want is the new method of the old plan. Let them try the new, and then they will know of the doctrine whether it be true or false.

EVENING SESSION, JULY 19, 1883.

Dr. J. W. Scarborough read a paper entitled "Lobelia Inflata as a Therapeutic Remedy."

This paper is unavoidably crowded out of this Number. In it Dr. Scarborough related a case in which severe and continued spasms of some kind followed the administration of ether as an anæsthetic, and which were relieved by large and repeated doses of Lobelia.—[EDITOR.]

*Dr. Luckey.*—I would like to ask the doctor whether there is any point where he would stop giving the remedy, in case no nausea was produced. He states that he gave it five or six times in increasing doses, the last dose being two tablespoonfuls. Is there not some limit at which you would stop in case there was no nausea, this side of death ?

*Dr. Scarborough.*—I would stop when the conditions requiring some relief ceased. If there were no spasmodic symptoms I would stop, certainly. With regard to the patient whose case I described,

I am positive she would not have lived two minutes the last time if I had not administered the final dose.

I have seen this medicine relax lock-jaw. When it was poured through the teeth the jaws sprung open immediately. It has an electric power that is possessed by nothing else that I know of in the whole range of materia medica. I will say that I am opposed to dosing and drugging and drastic purges. I could give my reasons for opposing physic; I have good reasons. But this medicine I speak of is not a physic. If there is something in the stomach that nature wants to remove, it may produce that effect, but when the system is cleared out you cannot physic with it; it will not act as a cathartic. In some of the works I have read, Lobelia is said to be a poison, which I do not believe.

*Dr. Hayhurst.*—Suppose that the two tablespoonfuls had not produced the desired effect, and you had gone on further, would it have come to a point where it would have been injurious to the patient?

*Dr. Scarborough.*—I should not have increased the dose beyond that, for I had given about four emetic doses. I started with ten drops, which gave relief, then in a short time the spasms returned, and I had to increase the dose.

*Dr. Robinson.*—Does it produce nausea on the patient?

*Dr. Scarborough.*—It did finally; but this patient was so far gone that the medicine could hardly produce any effect of that kind.

*Dr. Reading.*—At what stage did the spasmodic action appear?

*Dr. Scarborough.*—It was the very first thing that manifested itself.

*Dr. Reading.*—How did you administer the anæsthetic,—through a funnel?

*Dr. Scarborough.*—I moistened a sponge and placed it in a funnel-shaped napkin, held in the hand.

*Dr. Reading.*—I have noticed that if you wet the sponge pretty thoroughly in warm water it will, in a great many cases, prevent that spasmodic action you speak of, and an ounce of prevention is worth a hundred pounds of cure.

*Dr. Scarborough.*—A case like this was is very seldom seen.

*Dr. Reading.*—I have very often seen that condition when ether was given without the aid of a warm water sponge.

Dr. G. Carleton Brown read a paper entitled The Curse of Modern Civilization. (See page 526.)

*Dr. Atkinson.*—I think this is a very commendable effort. It is a recapitulation of some well-known facts with regard to the constitution of the berry of the wheat, which is the basis of our wheat flour, and the statements made in a mass way are well enough, but the execration of the carbonaceous portion of the berry is, I think, uncalled for, for the reason that fuel is as necessary to the production of steam as water is, and it is hardly fair to say that starch is a detrimental agent, inasmuch as it must be fermented in the human body until it shall have been converted into glucose, which is a form of sugar ; and it may be emulsified, and is supposed to contribute to the production of fat.

The problem is one that is too deep for us to take hold of seriously, unless we know more about it than we know in mass. Even the men who preside over the Health Boards and who have charge of the inspection of foods, are at loggerheads with regard to this question, as well as with regard to the subject of the paper we heard before it. Sometimes it is difficult for us to detect the real antecedent of the change that we speak of when nutrition is dealt with, for the reason that the condition of the body to be fed is one of the prime considerations in the administration of remedies. We need to be wide awake,—and the President spoke justly, for I was sleepy—to catch the true significance of the changes that are undergone in the digestory apparatus by these substances, which may be either foods, poisons or remedies. And here I want to warn you against the nomination of almost all the text-books, and to advise you to take in their stead this simple statement, that anything that is capable of acting upon the living human body has either to help or to arrest the nutrient changes that are going on, and may be, according to the demands and necessities of the body, either a food, a poison, or a remedy. And when we classify these substances as foods, poisons and remedies I would do it in this way : Lobelia is one, or the other, according to circumstances ; carbon is one, or the other, according to circumstances, and so on.

The outer husk of the berry of the wheat may be neither detrimental nor useless. You must have effete matter in sufficient quantity to make a bole, or mass of the rough pabulum, so that the vermicular motion of the intestines can pass it along, the absorbents taking up the fine pabulum, the excess and the effete matter being discharged at the terminal gut. It is the fine pabulum that feeds the tissues. We say pabulum. Some will say that means chyme, others will say it means chyle, and others that it means blood. It really means the product of the complete digestory process, after the food has gone through all the minutia of the churnings necessary to prepare it for appropriation.

Take certain quantities of oxygen and hydrogen, put them into a receiver and pass a current of electricity through them from the positive to the negative pole, and that does the courting and marrying both, and makes the hydrogen and oxygen cease to be gases and become a liquid which we call water. Then reverse the current, and they are gases again. These gases combine only in a definite proportion, which is two of hydrogen to one of oxygen; and when we shall have the combinations of the elements of food whittled down to as fine a point, we will then understand the processes by which this great mass of food material is emulsified, churned, mixed, carried through the chyliferous duct to the sub-clavian vein, and from there to the lungs, where it is calorified and converted into blood, to be distributed through the capillary system wherever it is needed. It is never pabulum until it has been oxygenated by passing through the lungs.

*Dr. G. Carleton Brown.*—I do not want to be understood as saying I would reject the starch, or carbon in the wheat. I think it would be just as bad, if we had only the phosphates without the carbon. If we have water without fuel we are in just as bad a fix as we are when we have fuel without water. But I do think that we need the phosphates above everything else at the present time, in view of the condition of our teeth; there at least the phosphates are greatly needed. Dr. Atkinson says if we have good, well organized teeth, we will have no need of dentists, and I say this is the road we will have to travel to get to that condition. If we burn the fuel without the water, if we eat the carbon of the wheat to the exclusion of the outside elements which contain the phosphates, we are bound to have a break down.

CLINIC EXHIBITS.

Dr. C. A. Timme, of Hoboken, N. J., demonstrated the use of Dr. R. Telschow's hydraulic press (designed to facilitate the stamping of metal plates) and a very small furnace for continuous gum work, which is heated by ordinary coal gas. The several steps to be taken in the use of the hydraulic press are as follows: An impression of plaster of Paris or impression material is coated with a thin film of oil or a solution of soap, a suitable rim of thick paper or thin sheet iron is put around the impression cup into which a compound of sulphur and plumbago and some other substances called Spence's metals is poured. (See August No. *Independent Practitioner*, page 415.) The melting of this metal must be done very slowly and under constant stirring. It melts at a temperature of about 280 F. when it is quite thick, but after stirring it for a minute after it has been removed from the fire, it becomes a comparatively thin liquid, and this is the proper time to pour it into the impression. The cast is then freed from the impression material and imbedded in the upper (bell) portion of the hydraulic press. If the alveolar ridge of the die is low and not overhanging, the metal plate can be pressed upon it without any other proceedure; if however the alveolar ridge is high, or very much overhanging, one zinc die is necessary upon which the folds of the plate can be hammered out, as the Spence metal can bear any amount of pressure but not the slightest tap with the hammer. A plate pressed by this press fits better than one stamped up in the old manner, and consequently requires no air chamber.

Dr. E. Slegel, of Reading, Pa., demonstrated the method of setting his new pivot tooth.

Dr. Wm. W. Evans, of Washington, D. C., restored for Dr. E. P. Brown, of Flushing, L. I., the mesial and cutting edge of the left upper central incisor, with gold which was introduced by the electro-magnetic mallet. Dr. E. P. Brown, after the operation, called attention to the neighboring tooth, the lateral, which contained a large filling of heavy gold foil made by Dr. Wm. H. Atkinson in the year 1868. The edges of the enamel had retained their normal color, and the filling appeared to be perfect in every respect.

Dr. Wm. W. Evans further exhibited a new apparatus for pressing or vulcanizing celluloid, zylonite, or rubber, and some beautifully made sets of artificial teeth mounted upon celluloid and zylonite.

Dr. Ryneer, of New York City, showed some samples of his new crown for molars and bicuspids.

Dr. Bonwill, of Philadelphia, exhibited his dental engine, new hand pieces, mechanical mallet, and all-porcelain tooth crowns. Such of them as are not entirely new have been, within the past year, so modified and improved that they present many new features, and they attracted the earnest attention of the dentists present.

Dr. Wm. H. Dibble, of Trenton, N. J., showed a lathe for grinding gum teeth, the new feature of this machine being a contrivance for holding the gum teeth in such a manner that a perfectly flat joint is invariably the result.

Mr. Geo. E. Hodge exhibited his well-known excellent hand piece for the dental engine.

The Wilmington Dental Manufacturing Co. had a well-selected stock of their teeth on exhibition.

The Redman Manufacturing Co., of Newark, N. J., showed a number of useful appliances, such as brackets, lathe heads, etc., all of which were exceedingly good for the low price.

Mr. R. S. Williams, of New York City, showed a great variety of his gold for filling teeth.

The S. S. White Dental Manufacturing Co. displayed their well-known dental materials and appliances, of which the new Cycloid chair deserves to be especially mentioned.

The Robinson Dental Manufacturing Co., of Jackson, Mich., exhibited their fibrous metallic filling.

Mr. L. D. Caulk, of Camden, Del., was also present to show his well-known varieties of filling materials.

Dr. A. N. Roussel, of Brooklyn, N. Y., showed his criterion gutta percha cylinders.

Dr. C. F. W. Bodecker, of New York City, regretted that he was unable to demonstrate microscopical specimens as announced, by means of the McIntosh Stereopticon, as the apparatus which was promised by Messrs. Tieman & Co., of New York, had not arrived.

